# The Avon Longitudinal Study of Parents and Children - a resource for COVID-19 research: questionnaire data capture July 2021 to December 2021, with a focus on long COVID

**DOI:** 10.12688/wellcomeopenres.19596.2

**Published:** 2023-09-07

**Authors:** Kate Northstone, Almudena Suarez-Perez, Sarah Matthews, Michael Crawford, Nicholas J. Timpson

**Affiliations:** 1 Department of Population Health Sciences, Bristol Medical School, University of Bristol, Bristol, England, BS8 2BN, UK; 2 MRC Integrative Epidemiology Unit, Department of Population Health Sciences, Bristol Medical School, University of Bristol, Bristol, England, BS8 2BN, UK

**Keywords:** ALSPAC, Children of the 90s, birth cohort study, COVID-19, coronavirus, mental health, long Covid, post-COVID syndrome

## Abstract

ALSPAC, the Avon Longitudinal Study of Parents and Children is a prospective population-based cohort study. Pregnant women were recruited in 1990-1992 and the study has followed them, their partners (Generation 0; G0) and their offspring (Generation 1; G1) for over 30 years. During the coronavirus 2019 (COVID-19) pandemic, ALSPAC deployed a series of online questionnaires to capture participant experiences during this unprecedented time. In July 2021, a fifth questionnaire was deployed which primarily focussed on the symptoms of long COVID, also known as post-COVID syndrome.

G0 and G1 participants were offered both online and paper questionnaires between 21 ^st^ July 2021 and 11 ^th^ December 2021. Of 21,138 invitations, 11,148 (52.8%) participants returned the questionnaire (4,763 original mothers [mean age 59.1 years], 2,074 original fathers/partners [mean age 62.0 years] and 4,311 offspring [mean age 29.0 years]). Of these 11,148 participants, 2835 (25.4%) had not completed any of the previous COVID-19 questionnaires, while 3480 (31.2%) had returned all four previous questionnaires.

In this questionnaire, 1077 participants (9.8%) reported a previous positive COVID-19 test over the course of the pandemic. Of these, 109 (1.0%) had received medical advice that they likely had COVID-19, and 838 (7.6%) suspected that they had had COVID-19. Almost a third of participants (n=796, 31.1%) reported possible long COVID (experiencing symptoms for at least 4 weeks), whilst 351 (13.7%) reported symptom duration of 12 weeks or more (post-COVID syndrome). G0 mothers were more likely to report a longer duration of symptoms compared to their partners and their children.

The fifth COVID-19 questionnaire deployed by ALSPAC and the data obtained from are described in this data note.

## Introduction

The coronavirus disease 2019 (COVID-19) pandemic has resulted in almost 7 million deaths across the globe, and the impact has profound consequences. At the time of writing (June 2023), the UK currently has no mandatory mitigations in place, with most nationwide restrictions eased some time ago. More detailed information on the COVID timeline in the UK can be viewed here. Despite the period of time that has passed since national mitigation was in place, the long-term impact of COVID-19 on the population’s physical and mental health is likely to be substantial. As are the social and financial consequences of the pandemic, which are likely to be evident for some time. It is therefore extremely important that ongoing population-based studies continue to prospectively measure the effects of these different dimensions.

Throughout the pandemic, the vast majority of people with COVID-19 infections were not admitted to hospital. However, debilitating ongoing physical and mental health symptoms affecting quality of life have been frequently reported. Long COVID, also known as post-COVID syndrome, is still being understood and currently the definition is unclear. The UK national Health Service (NHS) currently, defines long COVID in two ways: “Ongoing symptomatic COVID” – symptoms that carry on for 4 to 12 weeks and “Post-COVID syndrome” symptoms that persist for longer than 12 weeks ^1^. For reporting purposes the UK Office for National statistics defines long COVID as symptoms of COVID-19 lasting for at least 4 weeks after the onset of COVID-19 infection that cannot be explained by any other diagnosis ^2^. The latest statistics released by the UK Office for National Statistics estimate that in March 2023, 1.9 million people (2.9% of the population) had experienced long COVID ^2^.

The Avon Longitudinal Study of Parents and Children (ALSPAC; 3, 4), alongside a number of other longitudinal studies, has an exceptional opportunity to capture information on the symptoms of long COVID and help to define this new disease. ALSPAC is a unique three-generational study with 30 years of biological, genetic and phenotypic data already collected ^3– 7^. ALSPAC was able to react quickly to the pandemic and rapidly deployed a series of questionnaires to capture relevant data. In total, we have deployed six COVID-19 questionnaires across the original parent and offspring generations: 1) in April-May 2020 ^8^, 2) in May-July 2020 ^9^; 3) in October 2020 (with an antibody test) ^10^, 4) in November 2020-March 2021 ^11^, 5) in July-December 2021 and 6) in April-May 2022 (alongside an antibody test and online cognitive testing).

In this data note we briefly describe the data collected via our *fifth* COVID-19 questionnaire and how it can be used to contribute to our understanding of long COVID.

## Methods

### Ethics

Ethical approval for the study was obtained from the ALSPAC Ethics and Law Committee and the Local Research Ethics Committees. Written informed consent for the use of data collected via questionnaires and clinics was obtained from all participants following the recommendations of the ALSPAC Ethics and Law Committee at the time. Study participants have the right to withdraw their consent for elements of the study or from the study entirely at any time. Full details of the ALSPAC consent procedures are available on the study website.

### Setting

The Avon Longitudinal Study of Parents and Children (ALSPAC) is a unique multi-generational study which recruited pregnant women resident in Avon, South West England with expected dates of delivery 1 ^st^ April 1991 to 31 ^st^ December 1992 ^3, 4, 6, 7^. The initial cohort consisted of 14,541 pregnancies resulting in 14,062 live births and 13,988 children who were alive at 1 year of age. The women (G0 mothers), their partners (G0 partners) and their offspring (G1) have been followed up ever since. From the age of seven years onwards, the initial sample was bolstered with eligible cases who had originally failed to join the study and there were subsequently 14,901 children alive at 1 year of age following this further recruitment ^5^. Please note, the study website contains details of all the data that is available through a fully searchable data dictionary and variable search tool.

This was the first COVID-19 questionnaire where, we extended our online data collection to include all those participants for whom we did not have a valid email address (approximately half) and thus sent out postal questionnaires to those who had not contributed to the COVID-19 data collection effort previously (with the exception of the fourth COVID-19 questionnaire to G1– these questions were embedded within the wider ‘Life@28’ questionnaire as part of ALSPAC’s annual questionnaire strategy and thus could have been completed on paper). Participants were therefore invited to complete the fifth questionnaire with a physical invitation letter through the post if they did not have a valid email address.

The online version of the questionnaire was developed and deployed using REDCap (Research Electronic Data CAPture tools ^12^); a secure web application for building and managing online data collection exercises, hosted at the University of Bristol. Paper questionnaires were designed, scanned and verified using Teleform data capture software.

### Content design

Content for this questionnaire was designed in collaboration with a number of other longitudinal population studies forming the National Core Studies (NCS) Longitudinal health and Wealth work programme. The questionnaire was primarily developed in consultation with TwinsUK.

Content for this questionnaire was selected to address three needs:

1. The desire to capture information on the symptoms of long COVID and thus help to define this new condition.
2. The need to track changes in health and wellbeing over time using repeated measures. To address this, we repeated a panel of questions from our previous questionnaires (e.g., health symptoms and mental health). By repeating questions, we are also able to capture information about participants who have not completed previous COVID questionnaires.
3. As with our fourth questionnaire, the need to harmonize data collection with other cohorts to facilitate co-ordinated analyses as part of NCS.

The questionnaire captured information on the following (if the questions come from a standardised questionnaire, the source and reference has been provided in brackets; questions asked repeated from previous COVID-19 questionnaires have been noted in brackets as well):

A and B. Health
  • General health prior to the pandemic and notification of shielding
  • Symptoms of COVID-19 and negative control symptoms since the start of 2021 (symptoms repeated from Qs1-4)
  • New health conditions developed since the start of the pandemic
  • Diagnosis with COVID-19 (repeated from Qs1-4) and details about and duration of symptoms (if infected)
  • Other health-related issues (restricting physical activities, shortness of breath) and further questions about long COVID, including cognitive/concentration issues (repeated from Q4)

C. COVID Testing
  • Detailed questions on dates of testing, types of testing and results (not previously asked)

D. Vaccinations
  • Number received, type and dates
  • Potential side effects
  • Effects on menstruation ( online only)

E. Feelings
  • Depression assessed using the Edinburgh Postnatal Depression Scale (EPDS; 13; new but asked many times pre-pandemic in G0)
  • Depression assessed using the Short Moods and Feelings questionnaire (SMFQ; 14; repeated from Qs1-3)
  • Anxiety assessed using the General Anxiety Disorder-7 questionnaire (GAD7; 15; repeated from Qs1-3)

F. Sub-studies
  • Consent to be contacted about future sub-studies (new)

For the final questionnaire used, with the associated data dictionary (which includes frequencies of all variables that are available in each cohort) see *Extended data* (Timpson *et al*., 2022).

### Invitation and reminder strategy

Participants were not contacted if our administrative database record indicated that they were deceased, had withdrawn from the study, had declined further contact or had declined to complete questionnaires.

All G0 and G1 participants with an active email address or known home address, and who had not withdrawn from the study, were sent an invitation to complete the online questionnaire on 27 ^th^ July 2021. Non-responders were sent a reminder email to complete the online questionnaire. As part of our general questionnaire reminder strategy, a paper version of the questionnaire was then sent to those who had still not complete online or those participants for whom we did not have a valid email address. A total of 21,138 participants were invited to complete the questionnaire. All participants were offered an incentive (a £10 shopping voucher) for returning a questionnaire. In addition, we offered a prize draw (three prizes of £100) for those who completed their questionnaire by 30 ^th^ September 2021.

### Response rate

In total, 21,138 participants were invited to participate and questionnaires were completed by 11,148 participants (overall response rate of 52.7%). Table 1 summarises the response rate by cohort group. As with our previous COVID questionnaires, female participants were much more likely to respond than male participants. In addition, response rates were higher in G0 compared to G1. Of the 11,148 respondents, 2,835 (25%) had never completed a previous COVID questionnaire, 3,480 (32%) had returned all four previous COVID questionnaires (see Table 2).

**Table 1.** Number of participants who were eligible and who responded to the fifth COVID-19 questionnaire.

Cohort Group	Eligible ^1^	Responded to Q5 ^2^
G0 Mothers	9,271	4,763 (51.3%)
G0 Fathers/partners	3,137	2,074 (66.1%)
G1 Offspring daughters	4,889	2,833 (57.9%)
G1 Offspring sons	4,144	1,478 (35.7%)
**TOTAL**	**21,138**	**11,148 (52.7%)**

^1^Eligibility criteria (online and paper questionnaires): valid email and/or postal address, marked as contactable for questionnaires ^2^Proportions of those invited (i.e. eligible)

**Table 2.** Number of participants who responded to the fifth COVID-19 questionnaire and whether they completed previous ALSPAC COVID-19 questionnaires.

Previous COVID-19 questionnaires	G0 mothers	G0 fathers/ partners	G1 offspring	Total
No previous COVID-19 data	1540 (32%)	830 (40%)	465 (11%)	2835 (25%)
Returned at least one (but not all four) previous COVID-19 questionnaires	1676 (35%)	688 (33%)	2469 (57%)	4833 (43%)
Returned all four previous COVID-19 questionnaires	1547 (33%)	556 (27%)	1377 (32%)	3480 (32%)
**TOTAL**	**4763**	**2074**	**4311**	**11,148**

### Key results

Characteristics of responders according to key characteristics can be seen in Table 3. As with all previous questionnaires, those who responded were predominantly white and the majority had at least A-level qualifications (optional exams in the UK sat at the age of 18 years), with 50% of G0 mothers, 70% of G0 partners/fathers and 74% of G1 offspring in this category. G0 fathers/partners were 3 years older on average than G0 mothers (62 years vs 59 years), with G1 offspring having an average age of 29 years.

**Table 3.** Summary of key characteristics for those who responded to the fifth COVID questionnaire; n (%) for categorical variables or mean (sd) for continuous variables. The sample size for each characteristic is give in brackets after the % (for categorical variables) or sd (for continuous variables).

Key Characteristic	G0 Mothers	G1 Fathers/ partners	G1 Offspring
Age (years)	59.1 (4.5; *n* = 4,762)	62.0 (5.3; *n* = 2,077)	29.0 (0.65; *n* = 4,315 ^4^)
Latest BMI ^1^	26.5 (5.28; *n* = 3,450)	27.54 (4.05; *n* = 1,466)	24.6 (5.16; *n* = 3,491)
Latest Systolic BP ^1^	119.7 (14.28; *n* = 3,421)	132.8 (13.61; *n* = 1,480)	115.9 (11.08; *n* = 3,426)
Latest Diastolic BP ^1^	71.0 (9.59; *n* = 3,421)	77.3 (9.01; *n* = 1,480)	66.7 (7.73; *n* = 3,426)
Education level ^2^ ≥A level	2208 (49.6%; *n* = 4,452)	1,303 (66.7%; *n* = 1,954)	2,271 (73.9%; *n* = 3,072)
Ethnicity (from baseline ALSPAC) ^3^ White	4,348 (97.9%; *n* = 4,348)	1929 (98.4%; *n* = 1,961)	3,677 (96.2%; *n* = 3,677)

^1^Data taken from the most recent clinic that individual attended where available ^2^Data taken from pregnancy questionnaires for G0 and from most recent questionnaire for G1 where available ^3^Data taken from pregnancy questionnaires for all ^4^This sample size is lower than the total number of G1’s who returned a questionnaire ( *n* = 4,3xx) as data for four triplet/quadruplet pregnancies have been coded as missing in the release dataset for confidentiality reasons

Participants were asked whether they thought they have, or had ever had, COVID-19. Options were: ‘Yes, confirmed by a positive test’, ‘Yes, based on medical advice, ‘Yes, based on strong personal suspicions’, ‘Unsure’, ‘No’ or ‘Prefer not to answer’. 1,077 (9.8%) respondents reported that they had tested positive, 109 (1.0%) reported that COVID-19 was suggested based on medical advice and 838 (7.6%) believed they had COVID-19 due to their own suspicions. Table 4 summarises the responses to this question by cohort group. It is interesting to note that the proportion of participants who believe they had COVID-19 based on medical advice or their own suspicions barely changed from the fourth questionnaire (1.3% and 8.8% respectively), yet the proportion testing positive had almost tripled compared to the fourth questionnaire (3.5%).

**Table 4.** Participant response to whether they have had COVID-19 from the fifth COVID-19 questionnaire.

	G0 mothers	G0 partners/ fathers	G1 offspring	Total
Yes, confirmed by positive test	360 (7.6%)	143 (6.9%)	574 (13.4%)	1,077 (9.8%)
Yes, based on medical advice	61 (1.3%)	16 (0.8%)	93 (2.2%)	109 (1.0%)
Yes, own suspicions	340 (7.2%)	125 (6.1%)	373 (8.8%)	838 (7.6%)
No	3,671 (78.0%)	1,686 (81.6%)	2,983 (70.0%)	8,340 (75.9%)
Unsure/prefer not to answer	282 (5.9%)	96 (4.6%)	239 (5.6%)	617 (5.6%)

Participants were asked how long they had experienced their symptoms overall. They were given the options: ‘Less than 2 weeks’, ‘2–3 weeks’, ‘4–12 weeks’ or ‘More than 12 weeks’. Table 5 shows that overall 13.7% of participants had experienced symptoms for longer than 12 weeks whilst 17.4% experienced them for between 4 and 12 weeks. G0 mothers were more likely to report experiencing symptoms for longer compared to G0 partners and G1.

**Table 5.** Duration of COVID-19 symptoms.

	G0 mothers n=973	G0 partners/ fathers n=357	G1 offspring n=1232	Total n=2562
< 2 weeks	354 (36.4%)	164 (45.9%)	659 (53.5%)	1177 (45.9%)
2–3 weeks	244 (25.0%)	87 (24.4%)	258 (20.9%)	589 (23.0%)
4–12 weeks	206 (21.2%)	63 (17.6%)	176 (14.3%)	445 (17.4%)
12+ weeks	169 (17.4%)	43 (12.1%)	139 (11.3%)	351 (13.7%)

## Strengths and limitations of the data

We report a number of strengths in this dataset. Data has been collected in ALSPAC throughout the pandemic at multiple timepoints. Taken together with pre-pandemic baseline measures the study is in an excellent position to assess longitudinal changes in health and wellbeing and other aspects of the pandemic that may have a population level effect. This repeat questionnaire data can be supplemented by repeat serological measures ^10, 16^, linkage to national pillar testing ^17, 18^ and ongoing targeted, deep phenotyping studies.

In addition, ALSPAC led the Wellcome funded development of a core set of questions that could be used by any other longitudinal population based studies. This has led to collaboration with several UK studies providing several cross-cohort comparisons. A number of these have already been undertaken. These have focussed to date on mental health, population level disruption and the effects of employment change. We have already been able to demonstrate the short-term impact the pandemic has had on mental health during the first lockdown in April-June 2020 ^19^. Combined with 11 other studies, it has been shown that those participants with mental health pre-pandemic were more likely to experience disruptions to both healthcare and their financial situation ^20^. Meanwhile, healthcare disruption was more likely to be experienced by females, older people, those from ethnic minorities and lower social classes ^21^. A cross-cohort comparison with 10 other studies has shown that self-reported COVID-19 infection was associated with poorer mental health ^22^ and the effects of furlough (the UK government’s response providing 80% of salary to those employees who were unable to work during lockdown) on lifestyle changes ^23^.

A key limitation of this data collection is that it is possible that people with severe COVID-19 may be under-represented if they were too unwell to respond to questionnaires. It should be noted that the reverse may also be true; such that participants who have had COVID-19 (or believe that they have), may be more likely to engage with COVID-19 research given their personal experience. In addition, the response rate to all data collection exercises in ALSPAC are non-random with regard to age, sex and socio-economic status; this could potentially introduce some bias into analyses ^24^. However, this questionnaire received an excellent response rate and this was coincident with an offering on paper for the first time to both generations in ALSPAC. This also coincides with running the questionnaire for a longer period of time and offering a small monetary incentive (£10), which complies with ALSPAC’s usual procedures for a standard questionnaire. It is likely that these points in combination have led to a reduction in selection bias as evidenced by a decrease in the proportion of participants responding who had at least A level qualifications (optional exams taken at the age of 18 in the UK) at least in G0: 49.6% compared to 53.8% of mothers and 66.7% compared to 69.8% of partners completing COVID4). However the proportion of G1s with A levels remained the same (73.9% compared to 73.6%).

In summary, this ALSPAC data will hopefully contribute to an improved understanding of long COVID/post-COVID syndrome. In addition, repeat measures of aspects such as mental health will enable research to assess the ongoing impact of the pandemic.

## Figures and Tables

**Table 6.** Data from questions that will not be released until coded.

Question number	Question text
Section B	
B3a_other	Please tell us which of the following you have been told you have developed **since March 2020**. By this we mean a **new** problem that has started since March 2020. Other, please describe.
B18_other	What do you think would be most useful for people who continue to have symptoms **12 weeks after** their COVID-19 illness began? Other, please describe.
Section C	
C1_other	Have you **ever** had a swab test (or your nose and.or throat, or saliva) to see if you have COVID-19 at the time of testing? Yes, other reason, please describe.
Section D	
D3_other	What is the name of the **first (or only)** vaccine you received? Other, please describe.
D5_other	What is the name of the **second (or only)** vaccine you received? Other, please describe.
D7a_other	Did you experience any of the following side effects after your **first (or only)** vaccine injection? These usually develop within 48 hours of having the vaccine. Other, please describe.
D7b_other	Did you experience any of the following side effects after your **second (or only)** vaccine injection? These usually develop within 48 hours of having the vaccine. Other, please describe.

## Data Availability

ALSPAC data access is through a system of managed open access. The steps below highlight how to apply for access to the data included in this data note and all other ALSPAC data: 1. Please read the ALSPAC access policy ( www.bristol.ac.uk/media-library/sites/alspac/documents/researchers/data-access/ALSPAC_Access_Policy.pdf) which describes the process of accessing the data and samples in detail, and outlines the costs associated with doing so. 2. You may also find it useful to browse our fully searchable research proposals database ( https://proposals.epi.bristol.ac.uk/?q=proposalSummaries), which lists all research projects that have been approved since April 2011. 3. Please submit your research proposal ( https://proposals.epi.bristol.ac.uk/) for consideration by the ALSPAC Executive Committee. You will receive a response within 10 working days to advise you whether your proposal has been approved. Please note that a standard COVID-19 dataset will be made available at no charge (see description below); however, costs for required paperwork and any bespoke datasets required additional variables will apply. Open Science Framework: ALSPAC COVID-19 Data collections https://doi.org/10.17605/OSF.IO/6JR7E (Timpson *et al*., 2022) This project contains the following extended data: The final questionnaire (REDCap PDF) List of variable names and labels Associated data dictionary including frequencies of all variables that are available Data are available under the terms of the Creative Commons Attribution 4.0 International license (CC-BY 4.0).

## COVID-19 Questionnaire 5 Data File

Data from the fifth ALSPAC COVID-19 questionnaire is available in two ways.

1. A freely available standard set of data containing *all* participants together with key sociodemographic variables (where available) is available on request (see data availability section). This dataset also includes data obtained from the previous COVID questionnaires. Subject to the relevant paperwork being completed (costs may apply to cover administration) this dataset will be made freely available to any bona fide researcher requesting it. Variable names will follow the format *covid5_xxxx* where *xxxx* is a four-digit number. A full list of variables released is available here: https://osf.io/6jr7e/. Frequencies of variable and details of any coding/editing decisions and derived variables are also available in the data dictionary: https://osf.io/6jr7e/
2. Formal release files have been created for G0 mothers, G0 fathers and G1 participants in the usual way and now form part of the ALSPAC resource. These datasets (or sections therein) can be requested in the usual way. Variable names will replicate those in 1) above but as each variable in ALSPAC is uniquely defined we have added markers to denote the source of the variable. For example, in the fourth COVID-19 questionnaire dataset, the age of the participant at completion (in years) is denoted by *covid5_9650*. In the G0 mother’s dataset this will be denoted by *covid5m_9650*, for G0 fathers/partner this will be *covid5p_9650* and for the G1 generation it will be *covid5yp_9650*. Frequencies for all variables for each participant group are available in the data dictionary in the usual way ( http://www.bristol.ac.uk/alspac/external/documents/ALSPAC_Data_Dictionary.zip)
3. Text data and other potentially disclosive information will not be released until they have been coded appropriately. Table 6 describes the data that is withheld at the time of first release. Data will be incorporated back into both file sets as they become available.
