# Supplementary material for: The Avon Longitudinal Study of Parents and Children - a resource for COVID-19 research: questionnaire data capture July 2021 to December 2021, with a focus on long COVID
Source: Wellcome Open Res. 2023 Sep 7;8:292. Originally published 2023 Jul 10. [Version 2] doi: 10.12688/wellcomeopenres.19596.2 (PMC13069377; doi:10.12688/wellcomeopenres.19596.2)
Supplement: Peer review history

# Reviewer report: Reviewer response for version 2

**DOI:** 10.21956/wellcomeopenres.22181.r80473

**Published:** 2024-05-10

**Authors:** Darío Moreno-Agostino

**Affiliations:** 1 Centre for Longitudinal Studies, UCL Social Research Institute, University College London, London, England, UK

The authors have transparently addressed or responded to the previous reviewer's comments.

 One of the issues highlighted in the previous round seems to remain a problem (the link to “More detailed information on the COVID timeline in the UK” does not work https://www.bristol.ac.uk/alspac/researchers/wellcome-covid-19/; note that the same link is embedded later within the text  “core set of questions”).

 Beyond this, I perhaps have two small comments, but these don't require critical attention and I am only highlighting them in case they are helpful:

- Throughout the data note I was wondering what the target population of this data collection was. I think it would be interesting to include such information here so those interested in using the data can better understand whether it aims to be representative of any target population and if they may need to put in place any principled approaches to restore that representativeness due to the missing data not being completely random (e.g., by using non-response weights and whether these are provided with the dataset).
- It seems confusing to me that the data are described as "freely available" where accessing them seems to require the payment of an administration fee from most applicants (including "bona fide researchers", which is the reason I haven't been able to judge whether the datasets are "clearly presented in a usable and accessible format" and rated  this item with "Partly", as there is no "N/A" option).

Are sufficient details of methods and materials provided to allow replication by others?

Yes

Is the rationale for creating the dataset(s) clearly described?

Yes

Are the datasets clearly presented in a useable and accessible format?

Partly

Are the protocols appropriate and is the work technically sound?

Yes

Reviewer Expertise:

Social epidemiology, population-based research on mental health during COVID-19 pandemic

I confirm that I have read this submission and believe that I have an appropriate level of expertise to confirm that it is of an acceptable scientific standard.

# Response

**Published:** 2024-06-10

**Authors:** Kate Northstone

**Affiliations:** University of Bristol, UK

We thank the reviewer for taking the time to read our paper and for their helpful comments which we have addressed in an updated version. We have repeated the reviewer’s comments below and provide our response in italics.  

**Reviewer Comment:**

 “One of the issues highlighted in the previous round seems to remain a problem (the link to “More detailed information on the COVID timeline in the UK” does not work https://www.bristol.ac.uk/alspac/researchers/wellcome-covid-19/; note that the same link is embedded later within the text  “core set of questions”).”

***Author Response:*** *Thankyou for highlighting this, however, version 2 of the paper does have this correct:* *https://www.bristol.ac.uk/alspac/covid-19/wellcome-covid-19/*

**Reviewer Comment:**

*“*Throughout the data note I was wondering what the target population of this data collection was. I think it would be interesting to include such information here so those interested in using the data can better understand whether it aims to be representative of any target population and if they may need to put in place any principled approaches to restore that representativeness due to the missing data not being completely random (e.g., by using non-response weights and whether these are provided with the dataset).”

***Author Response:*** *This is an interesting point but difficult to respond to as we are aware that we have biased sample given the participants have been taking part in ALSPAC for >30 years. It is already stated in the discussion that response rates are non-random.  We do not provide non-response weights.*

**Reviewer Comment:**

 “It seems confusing to me that the data are described as "freely available" where accessing them seems to require the payment of an administration fee from most applicants (including "bona fide researchers", which is the reason I haven't been able to judge whether the datasets are "clearly presented in a usable and accessible format" and rated this item with "Partly", as there is no "N/A" option).”

***Author Response:*** *Thankyou for highlighting this and we apologise for any confusion here. To clarify, all data related to COVID-19 (collected from the 6 questionnaires in total that we administered during the pandemic) are available at no charge. However, if a researcher requires any additional data to be added to that dataset then our usual processing charges would apply. We have not changed the text to ensure that it remains consistent with all other data notes related to COVID-19 datasets, however, we can attempt to do this if the reviewer feels appropriate.*

# Reviewer report: Reviewer response for version 2

**DOI:** 10.21956/wellcomeopenres.22181.r66686

**Published:** 2023-10-17

**Authors:** Roberto Melotti

**Affiliations:** 1 Institute for Biomedicine, EURAC Research, Bolzano, Italy

I have no further remarks.

Are sufficient details of methods and materials provided to allow replication by others?

Yes

Is the rationale for creating the dataset(s) clearly described?

Yes

Are the datasets clearly presented in a useable and accessible format?

Yes

Are the protocols appropriate and is the work technically sound?

Yes

Reviewer Expertise:

Biostatistics, population-based COVID-19 research, public health

I confirm that I have read this submission and believe that I have an appropriate level of expertise to confirm that it is of an acceptable scientific standard.

# Reviewer report: Reviewer response for version 1

**DOI:** 10.21956/wellcomeopenres.21707.r62723

**Published:** 2023-08-17

**Authors:** Roberto Melotti

**Affiliations:** 1 Institute for Biomedicine, EURAC Research, Bolzano, Italy

Summary:

 This data note presents the fifth covid-related data collection from the multigenerational long-going and established resource of the ALSPAC birth-cohort. The specificities of these data collection are mainly two. 1) focus on symptoms which may refer to the syndrome known as long-covid or post-covid syndrome, 2) the extension of the target participants by ordinary mailed questionnaires to ALSPAC participants who had not filled any COVID-questionnaire before-hand.  

 While at random participation and scattered selection across time is an inevitable issue, this extra data collection further contributes to establishing population-based resources to study the effect of the pandemics with before-after designs in the long term, particularly on aspects of both physical and mental health. 

 I have one major and few minor comments, which I hope may improve the description of the resource.

Majors:

- Giving money incentives for participation in epidemiological studies is rather controversial in view of possible induction of selection bias. Please add some discussion on this topic and how this may have affected covid-related study participation, in particular.

Minors:

- Introduction, first paragraph: the link to the UK mitigation policies timeline is not working.
- Methods, content design, letter E. I'd entitle this item just 'Feelings' or 'self-mood', unless they are explicitly asking participants to report on the effect of the pandemic on their feelings.
- Methods, content design, last paragraph and sentence: *Extended data* (Timpson *et al*., 2022) unclear reference, please edit.
- In table 3, it is unclear how DBP and SBP are adding to the key characteristics of participants. e.g. do these values reflect different levels of hypertensive people compared with what you'd expect from a similar multi age-cohort. This could also aid indications for possible selection induced by either money incentives, else covid-related attitude, or else missing severe cases. Please comment in the discussion.
- In table 5, it would help to also report column count totals.
- References 10 and 16 appear as duplicates.
- Strengths and limitations of the data, second paragraph, first sentence: the link to the core set of questions doesn't work.

Are sufficient details of methods and materials provided to allow replication by others?

Yes

Is the rationale for creating the dataset(s) clearly described?

Yes

Are the datasets clearly presented in a useable and accessible format?

Yes

Are the protocols appropriate and is the work technically sound?

Yes

Reviewer Expertise:

Biostatistics, population-based COVID-19 research, public health

I confirm that I have read this submission and believe that I have an appropriate level of expertise to confirm that it is of an acceptable scientific standard, however I have significant reservations, as outlined above.

# Response

**Published:** 2023-09-06

**Authors:** Kate Northstone

**Affiliations:** University of Bristol, UK

We thank the reviewer for their constructive comments, and we hope that our revisions have addressed the critique. The reviewer’s comments are quoted below, followed by our responses in italics.   “Giving money incentives for participation in epidemiological studies is rather controversial in view of possible induction of selection bias. Please add some discussion on this topic and how this may have affected covid-related study participation, in particular.”

*Respectfully, we disagree with this point – there is evidence to suggest that monetary incentives improve selection bias in research studies  (e.g. see systematic reviews for participation in RCTs and for cohort studies respectively: Abdelazeem et al, 2022 doi: 10.1371/journal.pone.0267534 and Booker et al, 2011 doi: 10.1186/1471-2458-11-249). Aligned to this and compared to previous COVID_19 questionnaires, there is a decrease in the proportion of G0 mother and partner participants with A levels or higher (49.6% compared to 53.8% of mothers and 66.7% compared to 69.8% of partners completing COVID4) and G1 remaining virtually the same (73.9% compared to 73.6%).* *ALSPAC has been paying a small incentive to participants for many years, with full ethical approval. The increase in response rate for the 5th Covid questionnaire compared to the previous 4, will almost certainly be partly down to the incentive but also partly explained by the provision of a paper version and allowing the questionnaire to run for significantly longer than the previous Covid questionnaires.* *We have added discussion to this effect in the penultimate paragraph of the Strengths and Limitations section”*  

 “Introduction, first paragraph: the link to the UK mitigation policies timeline is not working.” *Thank you for highlighting this – we have amended this. *  

 “Methods, content design, letter E. I'd entitle this item just 'Feelings' or 'self-mood', unless they are explicitly asking participants to report on the effect of the pandemic on their feelings.”

*This has been changed to “Feelings” as suggested.*  

 “Methods, content design, last paragraph and sentence: Extended data (Timpson et al., 2022) unclear reference, please edit.” *This reference was added by the editorial office and points to the extended data section at the end of the paper, I don’t believe we can change this though will be happy to address this editorial change if needed.*

 “In table 3, it is unclear how DBP and SBP are adding to the key characteristics of participants. e.g. do these values reflect different levels of hypertensive people compared with what you'd expect from a similar multi age-cohort. This could also aid indications for possible selection induced by either money incentives, else covid-related attitude, or else missing severe cases. Please comment in the discussion.”

*We have consistently reported on blood pressure in the preceding 4 data notes as a simple indicator of physical health that is available for all cohorts. Compared to the data presented in data note 4 there is no difference in blood pressure on average in each cohort. As noted previously we do not believe that incentivising has introduced selection bias.*  

 “In table 5, it would help to also report column count totals.”

*We have added this as requested.*  

 “References 10 and 16 appear as duplicates.”

*Thank you for flagging this, this is an error. Reference 16 has been corrected.*  

 “Strengths and limitations of the data, second paragraph, first sentence: the link to the core set of questions doesn't work.”

*We have corrected the link.*
